# Endothelial YAP/TAZ Signaling in Angiogenesis and Tumor Vasculature

**DOI:** 10.3389/fonc.2020.612802

**Published:** 2021-02-04

**Authors:** Aukie Hooglugt, Miesje M. van der Stoel, Reinier A. Boon, Stephan Huveneers

**Affiliations:** ^1^ Department of Medical Biochemistry, Amsterdam Cardiovascular Sciences, Amsterdam UMC, University of Amsterdam, Amsterdam, Netherlands; ^2^ Department of Physiology, Amsterdam Cardiovascular Sciences, Amsterdam UMC, VU University Medical Center, Amsterdam, Netherlands; ^3^ German Center for Cardiovascular Research (DZHK), Partner Site Rhein-Main, Berlin, Germany; ^4^ Institute of Cardiovascular Regeneration, Goethe University, Frankfurt am Main, Germany

**Keywords:** yes-associated protein (YAP), TAZ, tumor vasculature, endothelium, mechanotransduction, cancer, Angiogenic therapy, tumor angiogenesis

## Abstract

Solid tumors are dependent on vascularization for their growth. The hypoxic, stiff, and pro-angiogenic tumor microenvironment induces angiogenesis, giving rise to an immature, proliferative, and permeable vasculature. The tumor vessels promote tumor metastasis and complicate delivery of anti-cancer therapies. In many types of tumors, YAP/TAZ activation is correlated with increased levels of angiogenesis. In addition, endothelial YAP/TAZ activation is important for the formation of new blood and lymphatic vessels during development. Oncogenic activation of YAP/TAZ in tumor cell growth and invasion has been studied in great detail, however the role of YAP/TAZ within the tumor endothelium remains insufficiently understood, which complicates therapeutic strategies aimed at targeting YAP/TAZ in cancer. Here, we overview the upstream signals from the tumor microenvironment that control endothelial YAP/TAZ activation and explore the role of their downstream targets in driving tumor angiogenesis. We further discuss the potential for anti-cancer treatments and vascular normalization strategies to improve tumor therapies.

## Introduction

It is estimated that solid tumors can grow to a size of approximately 2 mm^3^ without being vascularized (1). For further growth, tumors require blood vessels that deliver oxygen and nutrients. Tumors use several mechanisms for neovascularization, including angiogenesis, vessel co-option, vascular mimicry, trans-differentiation of cancer cells into endothelial cells (ECs), and through the recruitment of endothelial progenitor cells (2, 3). Angiogenesis, the formation of new vessels from pre-existing ones, is essential for tumor progression and growth and is promoted by pro-angiogenic signals secreted by the tumor cells and the tumor microenvironment (TME) (4, 5). The TME consists of cancer-associated fibroblast (CAFs), mesenchymal stromal cells, immune cells, ECs, as well as extracellular matrix (ECM) components, growth factors and cytokines (6–8). The tumor cells together with the TME, generate a hypoxic, acidic and inflammatory environment that further drives tumor angiogenesis, tumor growth and contributes to drug resistance (8, 9).

The tumor vasculature is morphologically different compared to the normal blood vessels (5, 9–11). Tumor angiogenesis gives rise to a dense and disorganized vessel network, in which the vessels are immature, dilated, hyperpermeable, and lack the support of pericytes or normal basement membrane. Tumor vessels are further characterized by irregular blood flow, which fails to supply sufficient oxygen and nutrients to the tumor tissue (12, 13). The tumor vessels contribute to malignancy by maintaining the hypoxic, acidic, and inflammatory environment, thereby fueling a vicious cycle that prevents normalization of the tumor vasculature and maintains a pro-metastatic environment (14, 15).

Targeting of tumor angiogenesis as treatment for cancer has been extensively investigated in pre-clinical and clinical settings. The first commercially available anti-angiogenic drug, Bevacizumab improves survival of patients with metastatic colorectal cancer by targeting of vascular endothelial growth factor (VEGF) (16). Currently, several VEGF-targeting FDA-approved drugs are used as anti-angiogenic cancer treatments, including for gastrointestinal cancer, glioblastoma, non-small lung carcinoma, breast cancer, and renal cancer (17, 18). Patient studies have shown increased survival after combining anti-VEGF therapy with chemotherapy (17, 19, 20). Unfortunately, long term administration of anti-VEGF treatments raises therapy resistance (21, 22) and major reductions in tumor blood vessels are not achieved, likely due to the activation of alternative neovascularization events in tumors (23, 24). Furthermore, pre-clinical *in vivo* studies have shown that after termination of anti-VEGF treatments, the tumor vessels rapidly return to an angiogenic and disorganized state (25).

Anti-angiogenic agents are often prescribed in high doses, which effectively leads to tumor vessel pruning and consequently decreases drug delivery to the TME (26). Moreover, local hypoxia in the TME, such as induced by vascular regression, enhances tumor invasiveness, chemo- and immunotherapy resistance, and metastasis (27). Hypoxia also induces expression of alternative angiogenic cytokines and compensatory mechanisms of neovascularization, further limiting the anti-angiogenic potential of anti-VEGF treatments (2, 28). Alternative angiogenic pathways are suspected to enhance tumor invasion and metastasis in response to anti-VEGF treatment (29).

Upon anti-angiogenic treatments, there is typically a short “window” during which vascular normalization is achieved, restoring normal blood vessel function and reducing hypoxia in the TME (21, 30–32). During this time frame, radiation- and immunotherapies were found to be most effective (33, 34). Because high doses and long-term treatment of anti-angiogenic drugs promote hypoxia in the tumor tissue, it is thought that lowering of drug dosage may reduce the levels of angiogenic factors and normalize the tumor vasculature accordingly (30). Experimental tumorigenesis studies using low doses or short-term treatments of anti-angiogenic drugs, indeed observed increased functional blood vessels, improved immunotherapy efficacy, and reduced metastatic activity of tumor cells (26, 35, 36). The discovery of additional therapeutic strategies are pursued to try to overcome anti-angiogenic resistance and to better control normalization of tumor vessels (37, 38).

The oncogene Yes-associated protein (YAP) and its paralogue Transcriptional Co-Activator With PDZ-binding Motif (TAZ or WWTR1) have been considered as attractive pharmacological targets, as they are highly activated in many forms of cancers and contribute to tumor growth and invasion (39). YAP/TAZ are also well known for their regulatory role during physiological and developmental angiogenesis and have recently gained attention in the context of endothelial-driven tumor angiogenesis (40–42). In this review we aim to understand how YAP/TAZ signaling affects the (tumor) endothelium. We will further discuss the potential mechanisms of YAP/TAZ activation by the TME and the downstream transcriptional program of YAP/TAZ that controls angiogenesis.

## Molecular Regulation of YAP/TAZ Activity

In general, in normal quiescent adherent cells YAP/TAZ are inhibited by the Hippo pathway and located in the cytoplasm. Upon various activating signals YAP/TAZ translocate toward the nucleus and act as transcriptional co-factors for the regulation of tissue homeostasis and organ growth (43, 44). In addition, YAP/TAZ respond to mechanical stimuli derived from cell spreading, contact inhibition, cytoskeletal contractility, ECM stiffness and fluid shear forces (45, 46).

The Hippo signaling pathway consists of a phosphorylation cascade with several effectors. Serine/threonine kinases 3 and 4 (STK3 and STK4, also called MST1/2) interact with the scaffolding protein Salvador family WW-domain-containing-protein-1 (SAV1). If Hippo signaling is turned “on”, the MST-SAV1 complex phosphorylates MOB kinase activator 1A and 1B (MOB1A and MOB1B). This leads to an interaction of MOB1A and MOB1B with large tumor suppressor kinase 1 and 2 (LATS1/2) (47, 48). Once in complex with MOB1A/1B, LATS1/2 become autophosphorylated and phosphorylated by MST1/2 (47, 49). In turn, active LATS1/2 kinases phosphorylate YAP on 5 serine residues (S61, S109, S127, S164, and S381) and TAZ on 4 serine residues (S66, S89, S117, S311) (50). Serine phosphorylated YAP/TAZ bind to 14-3-3 proteins, which sequesters the proteins in the cytoplasm or targets YAP/TAZ for ubiquitin-mediated proteasomal degradation (51, 52). Alternatively, YAP/TAZ can be sequestered in the cytoplasm by Angiomotin (AMOT) proteins that interact with YAP/TAZ or Hippo pathway effectors. If Hippo signaling is turned “off”, YAP/TAZ act as transcriptional co-activators in the nucleus, where they primarily interact with TEA domain family member (TEAD) transcriptional factors to regulate genes involved in proliferation, migration and survival (53). The Hippo pathway crosstalks with major signaling routes that control tissue remodeling and growth, including the Wnt/β-catenin, TGFβ, and Notch pathways (54–56).

YAP/TAZ are also activated in various force-dependent manners (43, 45, 51). Upon cytoskeletal-driven cellular adaptations, such as during ECM stiffening, shear stress sensing or upon G-protein-coupled receptor (GPCR) signaling, the AMOT proteins enhance their interaction with F-actin, allowing YAP/TAZ to translocate toward the nucleus (57–60). ECM stiffening also remodels the integrin-based focal adhesions (FA). Cell adhesion promotes activation of Focal Adhesion Kinase (FAK) and SRC tyrosine kinases, that impinge on the Hippo pathway through direct activation of YAP/TAZ and inhibition of LATS1/2 and MOB1 through FAK/Rac and SRC/PI3K signaling (51, 61). The stiffness-sensing integrin receptors transduce forces to the cytoskeleton-anchored nucleus, opening the nuclear pores and driving YAP/TAZ nuclear translocation (46). Mechanical forces at cell-cell junctions also control YAP/TAZ, as strain on epithelial monolayers induce β-catenin and YAP1 nuclear localization (62, 63). Moreover, high tension inferred at the junctional cadherin-catenin complex, triggers the interaction of α-catenin with TRIP6 and LIMD1, which recruit LATS1/2 to the junctions and inhibit their kinase activity, leading to nuclear translocation of YAP/TAZ (64–66). The various Hippo and mechanotransduction pathways that regulate YAP/TAZ have been elegantly discussed previously (45, 63, 67, 68). These molecular pathways have been investigated in great detail in normal epithelia or tumor cells and are expected to be responsible for YAP/TAZ regulation in the endothelium as well (45).

## The Role of YAP/TAZ in Developmental Angiogenesis

Angiogenesis is driven by endothelial proliferation, collective cell migration, and cellular rearrangements (69–71). Angiogenic stimuli, such as VEGF-A and FGF2, activate the ECs to promote the formation of endothelial tip cells that migrate toward the angiogenic cue. Tip cells are followed by proliferative endothelial stalk cells, which shape the developing sprouts and the vascular lumen (2, 72, 73). The tip and stalk cell rearrangements are regulated through feedback loops between VEGF and Dll4/Notch signaling (74).

YAP/TAZ are activated in the sprouting ECs of the developing vasculature in the mouse retina (75). YAP/TAZ are expressed in the entire retinal vasculature, but they reside in the endothelial cytoplasm in the central vascular region, while YAP/TAZ are mainly nuclear in sprouting ECs and the remodeling vascular plexus (76–78). Especially, TAZ nuclear localization is prominent in spouting ECs of the developing retinal vasculature (76, 77). Importantly, the activation of YAP/TAZ in ECs is crucial for angiogenesis (75, 77). The nuclear translocation of endothelial YAP/TAZ is regulated by VEGF signaling, VE-cadherin-based adherens junctions, and cytoskeletal remodeling (45, 75, 79, 80). The VE-cadherin complex sequesters YAP/TAZ and (force-dependent) remodeling of the cell-cell junctions leads to YAP/TAZ activation (81, 82). VEGF-A signaling stimulates YAP/TAZ through cytoskeletal remodeling and inactivation of the Hippo effectors LATS1/2 (79). In turn, active endothelial YAP/TAZ induce a downstream transcriptional program which regulates proliferation, actin cytoskeleton contractility, cell adhesion, and collective cell migration (76, 77, 79, 80, 83).

The importance of YAP/TAZ function for vascular development has been studied by several groups using (inducible) endothelial-specific YAP/TAZ double knock out mouse models (76, 77, 79, 83). Endothelial-specific depletion of YAP/TAZ reduces the number of tip cells and angiogenic sprouts, and leads to excessive vessel crossing in the developing vasculature of the mouse retina (76, 77, 79, 83). Moreover, once the vasculature in the YAP/TAZ endothelial-specific knockout mice makes it to the stage of larger vessels, the vessels turn out to be leaky due to perturbation of endothelial cell-cell junctions (76, 77). Interestingly, knocking out only the YAP or TAZ genes from the endothelium resulted in mild vascular defects, indicating that YAP and TAZ have redundant functions and can compensate for each other in the endothelium (77).

Endothelial-specific overexpression of YAP or TAZ induced retinal vessel growth through increased angiogenic sprouting (77, 84). Interestingly, endothelial-specific YAP overexpression did not affect the vasculature of quiescent tissue in adult mice (84). Constitutive activation of YAP/TAZ, induced by knockout of LATS kinases or overexpression of an active mutant of YAP or TAZ, resulted in endothelial hypersprouting *in vivo* (77, 83). *In vitro* it was found that overexpression of an active form of YAP promotes hypersprouting *via* the angiogenic growth factor angiopoietin-2 (Ang2) signaling (75).

In agreement with the knockout mouse models that demonstrate an important role for YAP/TAZ in vascular development, depleting YAP/TAZ from zebrafish resulted in embryonic lethality due to severe developmental and vascular malformations (85, 86). YAP1 null mutant zebrafish showed a drastic reduction in transcriptional activity of TEAD2, while in TAZ null mutant zebrafish TEAD2 transcriptional activity was unaffected (86), suggesting that YAP is the major transcriptional regulator for vascular development in zebrafish. YAP1 null mutant zebrafish showed increased vessel regression and lumen stenosis, suggesting an important role for YAP1 in lumen maintenance in response to blood flow (86). Moreover, truncation of the cranial and ocular vasculature is observed (85). By contrast, TAZ null mutants did not display clear vascular defects (85). Transgenic mutant zebrafish in which the binding of the YAP/TAZ-TEAD complex to the DNA has been prevented, display altered vascular remodeling (87). Interestingly, expression of constitutively active YAP (YAP-5SA), TAZ (TAZ-4SA), or TEAD mutants initially promote vessel sprouting, but the sprouts fail to anastomose or stabilize at later stages (85). In summary, the regulation of endothelial YAP/TAZ activity is critical during vascular development and both the down- and upregulation of YAP/TAZ activity leads to aberrant sprouting angiogenesis and blood vessel formation.

**Figure 1:**
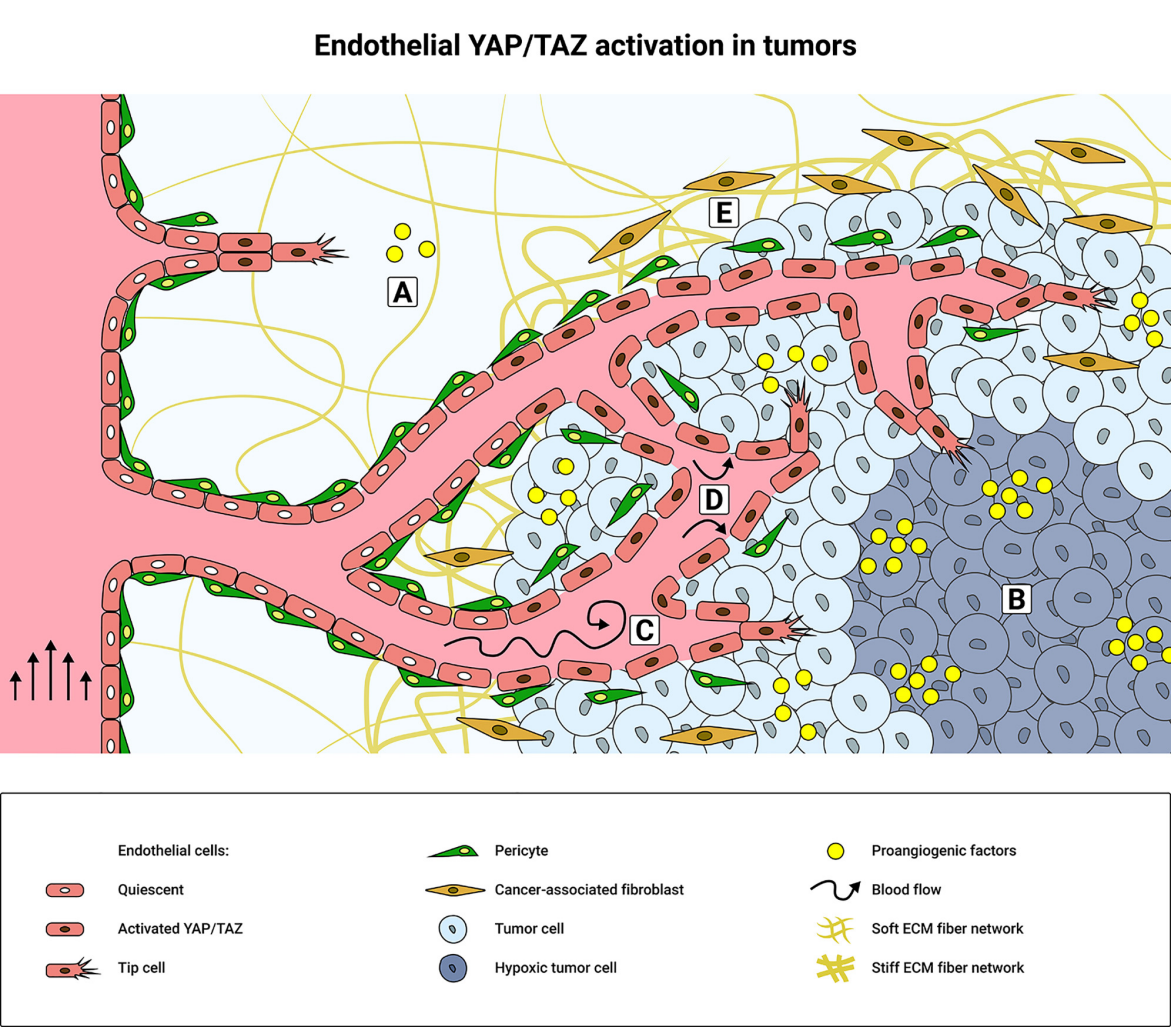
Schematic overview of tumor microenvironment factors that promote endothelial YAP/TAZ activity. **(A)** During physiological angiogenesis, pro-angiogenic cytokines and growth factors activate YAP/TAZ signaling in endothelial cells (ECs) through GPCRs and RTKs, such as VEGFR2 or Tie2. **(B)** Hypoxia induces secretion of pro-angiogenic factors to promote tumor angiogenesis and consequently YAP/TAZ activity in the tumor microenvironment (TME). Elevated levels of pro-angiogenic factors promote the formation of a dense and disorganized tumor vasculature, resulting in disturbed blood flow patterns **(C)** and increased interstitial fluid pressure **(D)** that mechanically activate YAP/TAZ. The integrity of the tumor endothelium is weakened due to decreased cell-cell interactions between the ECs and/or pericytes. YAP/TAZ are further mechanically activated in the TME by ECM stiffening **(E)**, which is promoted by the cancer-associated fibroblasts (CAFs).

After new blood vessels are formed during development, angiogenic growth factor levels drop and vessels mature through the stabilization of cell-cell junctions and the recruitment of mural pericytes. In mature vessels, ECs are quiescent (88–90) and endothelial YAP/TAZ are inactivated (79). During wound healing in physiological and pathological conditions, YAP/TAZ are activated on demand to induce angiogenesis (53). The TME is somewhat comparable to the tissue of an inflamed wound (91), but in tumors endothelial YAP/TAZ remain activated and its vasculature does not evolve into a mature state (79) (**Figure 1**).

## YAP/TAZ Activation in Tumor Angiogenesis

Many types of cancer are accompanied by increased levels and activity of YAP/TAZ, including breast, pancreatic, liver and colorectal cancer (39, 92–94). Increased expression or activation of YAP/TAZ in cancer is associated with poor prognosis and reduced survival (93, 95). Increased YAP/TAZ levels are often observed both in tumor and stromal cells, including the CAFs, ECs, immune cells, and pericytes (96–99). YAP/TAZ activation in the TME promotes tumor growth, metastasis and angiogenesis (95, 97–99). For instance, in glioblastoma, high expression of TAZ in tumor endothelium is correlated with increased blood vessel density and tumor malignancy (100). There is an intricate connection between YAP/TAZ activation in the TME and the tumor vasculature.

Oncogenic activation of YAP/TAZ in tumor cells drives the ECs toward a pro-angiogenic state (**Figure 1**). Conditioned medium from (YAP positive) breast cancer cells induced endothelial YAP activation, which in turn promoted tumor angiogenesis (101). Also, ECs treated with conditioned medium from cholangiocarcinoma cells containing a constitutively active YAP mutation (YAP S127A), showed increased tube formation capacity *in vitro* (102), suggesting enhanced endothelial activity. Moreover, YAP activation in mesenchymal stromal cells has been shown to enhance the crosstalk between gastric cancer cells and the tumor endothelium (103). Importantly, transplantations of Lewis Lung carcinoma allografts in transgenic mice with endothelial-specific YAP overexpression, resulted in an increase in tumor size and tumor vasculature (84). Vice versa, YAP knockdown in renal cell carcinoma, inhibited the angiogenic capacity of ECs *via* paracrine VEGF signaling (104). Overall, these findings emphasize the critical involvement of endothelial YAP/TAZ signaling during tumor angiogenesis following the interplay between tumor tissue and the ECs. Of note, YAP/TAZ activation does not always increase tumor angiogenesis and differences between tumor types should be considered. For instance, in angiosarcoma, a rare type of cancer derived from the vasculature, inhibition of PECAM-1 raised YAP levels, but decreased the tubulogenic potential of the angiosarcoma cells (105).

## Tumor Microenvironmental Factors That Activate YAP/TAZ

In solid tumors many microenvironmental properties have changed compared to in healthy tissue, for example increased interstitial fluid pressure (IFP), inflammation and ECM stiffness. These TME properties are key drivers of YAP/TAZ and promote the immature characteristics of the tumor vasculature (43, 106) (**Figure 1**).

### Hypoxia

One of the prominent angiogenic features of solid tumors is their hypoxic condition. Hypoxia stabilizes hypoxia inducible transcription factor 1α (HIF1α) in tumor cells, initiating the transcription and secretion of pro-angiogenic factors, such as VEGF and Ang2 (107). Also in ECs, HIF1α promotes the transcription of autocrine pro-angiogenic molecules and matrix metalloproteases (MMPs) (40). Importantly, the endothelial-specific depletion of HIF1α resulted in reduced tumor growth and angiogenesis in experimental Lewis lung carcinoma (108). The onset of hypoxia in the retinal vasculature is known to inhibit Hippo pathway effectors and activates endothelial YAP (109). In turn, YAP is able to interact with HIF1α to sustain HIF1α signaling (110). In hypoxic colorectal cancer cells, HIF1α induces the transcription of GPCR5A, which in turn activates YAP to promote cell survival (111). Of note, in hepatocellular carcinoma cells, hypoxia was shown to activate YAP through a HIF1α independent manner (112). The intricate crosstalk between YAP/TAZ and HIF1α signaling in cancer has recently been overviewed (113).

**Figure 2:**
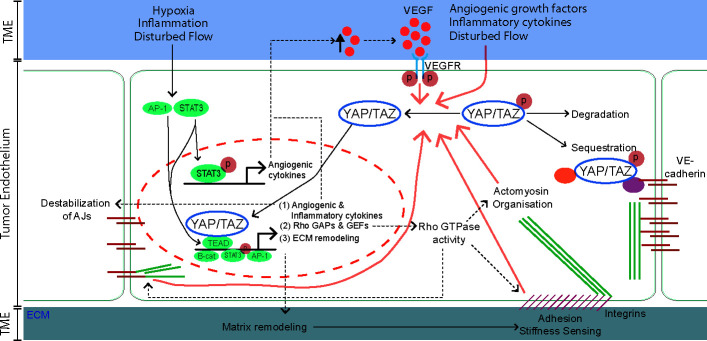
The tumor microenvironment (TME) and downstream transcriptional targets of YAP/TAZ engage in a positive feedback program that sustains YAP/TAZ activity in the endothelium. In quiescent endothelial cells YAP/TAZ are kept inactive and sequestered in the cytoplasm *via* interaction with 14-3-3 proteins, Angiomotin (AMOT) proteins or the VE-cadherin complex. Alternatively, inactive YAP/TAZ can be targeted for ubiquitin-mediated degradation. External cues from the tumor microenvironment (TME), including pro-angiogenic growth factors, inflammatory cytokines, stiff extracellular matrix (ECM), hypoxia, and disturbed blood flow activate YAP/TAZ leading to their translocation to the nucleus (**Figure 1**). In parallel, multiple TME factors activate the transcription factors STAT3 and AP-1. Within the nucleus, YAP/TAZ interacts with several transcription factors, most notably the TEA domain family members (TEADs), but also with STAT3 and β-catenin to induce transcription of downstream target genes. Activated YAP/TAZ induce the expression of angiogenic and inflammatory cytokines, Rho GAPs and GEFs and extracellular matrix (ECM) remodeling proteins that engage in a positive feedback system that sustains YAP/TAZ activity. (1) angiogenic and inflammatory cytokines maintain the pro-angiogenic and inflammatory TME. Moreover, the angiogenic effectors of YAP/TAZ (such as Ang2 and VEGF) destabilize VE-cadherin-based adherens junctions (AJs), further promoting the activity of YAP/TAZ. (2) Rho GAPs and GEFs control the level of Rho-GTPase activities, crucial switches that organize the actomyosin cytoskeleton, AJs and integrin-based focal adhesions (FA). In turn, the FAs and actomyosin cytoskeleton transduce forces from stiff ECM and maintain the nuclear translocation of YAP/TAZ. (3) ECM remodeling proteins are secreted and remodel the TME in favor of YAP/TAZ activity.

Hypoxia also induces the activation of Signal Transducer and Activator of Transcription-3 (STAT3), which forms a complex with YAP in ECs to drive expression of angiogenic factors VEGF and Ang2 (84, 109) (**Figure 2**). Furthermore, the transcription factor SNAIL is a direct target of HIF1α, and hypoxia-induced expression of SNAIL promotes endothelial to mesenchymal transition (EndoMT) (114). YAP has been shown to induce EndoMT during cardiac development by upregulating expression of SNAIL (115). YAP activation potentially promotes hypoxia-induced EndoMT in the TME by facilitating SNAIL expression. Taken together, the hypoxic conditions within tumors activate YAP/TAZ-dependent programs that promote tumor vascularization. Notably, the described crosstalk between YAP/TAZ and HIF1α may differ between tissues, because in the hypoxic environment of the bone marrow, endothelial YAP/TAZ function to inhibit angiogenesis (116).

### Angiogenic Growth Factors and Cytokines

A second important TME property that promotes endothelial YAP/TAZ activity and angiogenesis is the increased presence of angiogenic and inflammatory cytokines (i.e., VEGF, TGFβ and TNFα). Key is the (hypoxia-driven) VEGF production by tumor cells, ECs, and CAFs (117, 118). VEGF interaction with VEGF receptor 2 (VEGFR2) induces downstream signaling toward SRC kinases, PI3K/Akt, and MEK/ERK. These signaling pathways inactivate the Hippo pathway effectors MST1/2 and LATS1/2, leading to activation of endothelial YAP/TAZ (79, 119). The stimulation of ECs by VEGF also remodels the actin cytoskeleton and endothelial cell-cell junctions (120, 121), which further modulates YAP/TAZ activity (79). Actomyosin contractility is a well-established regulator of YAP/TAZ activity in a variety of cell types, acting directly on YAP/TAZ or *via* Hippo pathway effectors (45, 46, 122). In summary, VEGF-VEGFR2 signaling can induce endothelial YAP activation through various pathways, directly by interrupting the Hippo signaling cascade, or indirectly by its effect on cell-cell junctions and the actin cytoskeleton. Similar mechanisms may take place in the tumor endothelium.

Another important growth factor that affects YAP/TAZ signaling in tumors is TGFβ. Hypoxia together with TGFβ-mediated SMAD transcriptional activation induces complex formation between Zyxin, LATS2 and SIAH2 (123). The latter being an E3 ligase that mediates LATS2 ubiquitination and degradation (123). Furthermore, SMAD2 can interact with YAP *via* the RASS1FA scaffold protein, which helps to retain the SMAD2/YAP complex in the cytoplasm in quiescent cells (124). TGFβ induces the degradation of RASS1FA, which enables the SMAD2/YAP complex to translocate to the nucleus, leading to transcription of their target genes (124). YAP plays a crucial role in the nuclear translocation of SMADs (125). Most of these molecular mechanisms have been studied in epithelial or tumor cells. Yet, the tumor ECs express high levels of Endoglin (126), a BMP9 receptor that is part of the TGFβ receptor complex and an important receptor for endothelial migration and angiogenesis (127–129). BMP9-endoglin signaling induces YAP/SMAD nuclear translocation driving the expression of inflammatory genes in ECs (130), implying that endothelial YAP-SMAD may regulate tumor angiogenesis. Taken together, there are multiple angiogenic growth factors and cytokines within the TME that control YAP/TAZ and contribute to the immature and proliferative tumor vasculature.

### Tumor Microenvironment Stiffness

Overall, solid tumors are stiffer in comparison to normal tissue (106). The stiffening is caused by increased collagen deposition and cross-linking (131). In tumors, ECM stiffening promotes sprouting angiogenesis and gives rise to a dense and hyperpermeable vasculature (106). Integrin adhesion receptors sense the increased ECM stiffness and induce the assembly of integrin-based FAs (132, 133). In turn, FAK/SRC signaling downstream of integrins inhibits LATS1/2 and activates YAP/TAZ (51, 134–136). The activation of endothelial FAK through phosphorylation of its autophosphorylated Y397 residue is crucial for tumor angiogenesis and tumor progression (137). Interestingly, targeting of endothelial FAK activity improved the efficacy of DNA-damaging chemotherapeutics, providing proof-of-principle for normalization of tumor vasculature as an adjuvant approach in cancer therapies (138). Importantly, integrin-mediated stiffness-sensing also activates YAP/TAZ independently of the Hippo pathway through activation of Rho-GTPases and actomyosin contractility (45, 46). YAP/TAZ activation, in turn, regulates FA turnover (139), a feedback mechanism that is crucial for endothelial collective migration and angiogenic sprouting (80, 140). Taken together, it is likely that direct stiffness-sensing through the tumor endothelium promotes endothelial YAP/TAZ activation and tumor angiogenesis. To prove this concept more experimental work is needed.

In addition, TME stiffening and tumor cell contractility promote MMP activity (141). MMP activity and subsequent matrix degradation controls endothelial sprouting through the stiff ECM (131). Vice versa, inhibition of lysyl oxidase, a matrix cross-linking enzyme involved in tumor stiffening, was shown to reduce the tumor stiffness and tumor vasculature in a mouse mammary tumor model (131). It was recently reported that metastasis-associated fibroblasts promote TME stiffening and angiogenesis in particular in liver metastases from colorectal cancer (142). Intriguingly, inhibition of renin-angiotensin signaling in combination with the anti-VEGF drug Bevacizumab, inactivated tumor endothelial YAP and reduced the fibroblast-induced metastatic TME stiffness and tumor vasculature (142). Moreover, the activation of YAP in CAFs is known to increase ECM stiffening, tumor cell invasion and tumor angiogenesis (97). Thus CAFs participate in a self-sustaining YAP-dependent feed forward loop that aggravates tumorigenesis, through a mechanism in which tumor stiffening and angiogenesis take central roles (**Figure 1**).

### Interstitial Fluid Pressure and Blood Flow

Tumor vessels are disorganized and hyperpermeable, which increases interstitial fluid pressure (IFP) and leads to disturbed blood flow (143), altogether hindering drug delivery to tumors (144). The high level of IFP also stretches the blood vessels and promotes metastasis (145–148). Mechanical stretching of tumor stromal cells, such as fibroblasts and ECs, is a well-known driver of YAP/TAZ nuclear translocation (46, 77, 149, 150). Consequently, IFP likely activates endothelial YAP/TAZ signaling in the TME. The tumor vasculature is also poorly perfused and the blood flow is often turbulent (13, 151). Disturbed flow patterns activate endothelial YAP/TAZ and trigger proliferative and inflammatory responses *in vitro* (152–154). In the mouse aortic arch, a vascular area exposed to disturbed blood flow, YAP phosphorylation was lower and its nuclear localization higher, as in comparison to the thoracic aorta, an area exposed to laminar blood flow (153). Consequently, disturbed haemodynamics activate endothelial YAP/TAZ and we speculate that they sustain YAP/TAZ signaling in the tumor vasculature.

In summary, the pro-angiogenic, inflammatory, hypoxic, and stiff TME activates endothelial YAP/TAZ and promotes tumor angiogenesis. A major challenge is to tackle the essential events that sustain this oncogenic vascular environment. Investing in pre-clinical engineered tumor vascular models in which the TME cues can be manipulated in a defined manner are expected to greatly propel research aimed at developing anti-angiogenic therapies for cancer (155).

## Downstream Effectors of YAP/TAZ in Tumor Angiogenesis

To date, the mechanisms through which YAP/TAZ control (tumor) angiogenesis remains unclear. Active YAP/TAZ act as co-factors and bind to transcription factors to modulate gene expression (156). YAP/TAZ both contain a TEAD-binding domain and one WW (TAZ) or two WW domains (YAP) that mediate the interaction with transcription factors (157). The interaction of YAP/TAZ with the transcriptional enhancer factor domain (TEAD) family has been considered as the primary axis through which YAP/TAZ regulate transcription. TEAD-dependent transcription include most of the classical YAP/TAZ target genes, including CTGF, CYR61, and ANKRD1 (158–160). The YAP-TEAD complex is important for tumorigenesis: mutating the TEAD binding domain in YAP suppresses its oncogenic capacity in cancer cells (159, 161). The ability of tumor tissue to promote tumor angiogenesis through expression of angiogenic factors occurs in a TEAD-dependent manner (102, 162). Furthermore, pharmacological inhibition of the YAP-TEAD complex with verteporfin suppresses tumor growth in pancreatic cancer, by inhibiting the proliferation of pancreatic ductal adenocarcinoma cells and through inhibition of the angiogenic activity of associated tumor ECs (162).

The WW domains of YAP/TAZ bind to proline-rich sequences such as the PPXY motif, found in a variety of transcription factors, and they mediate the interaction with SMADs, AMOTs, ErbB4, β-catenin, RUNXs, and p73 (163). Mutations in the WW domain perturb YAP-controlled transcriptional programs and reduce its oncogenic capacity (161). While not yet investigated directly, the WW domains of YAP/TAZ are likely to modulate tumor angiogenesis, as YAP/TAZ WW-binding proteins, such as SMADs and AMOTs, have readily been linked to angiogenesis (164, 165).

Promoting gene expression through TEAD-binding or the WW domains are not the only mechanisms through which YAP/TAZ may regulate tumor angiogenesis. For instance TBX5, a transcription factor that lacks a PPXY-motif, can interact with YAP/TAZ *via* its carboxyl-terminus (166). Moreover, SMAD2/3 can bind the coiled-coil region of YAP/TAZ (167). Multiple YAP/TAZ interactors can synergize to promote gene expression. For instance, ErbB4 binds to YAP through the WW domain (168), which promotes the binding of TEAD to YAP. In this way, ErbB4 regulates the expression of the canonical YAP/TAZ-TEAD targets, including CTGF, CYR61, and ANKRD1 (169). AP-1 is a transcription factor present in most of YAP/TAZ-TEAD genomic binding sites and its presence greatly enhances oncogenic growth induced by active YAP/TAZ. Conversely, AP-1 inactivation inhibits YAP/TAZ-driven proliferation and tumorigenesis (170, 171). AP-1 does not directly bind to YAP/TAZ, but it controls TEAD-dependent gene expression in a cis-regulatory fashion (170–172) (**Figure 2**). Interestingly, TRPS1 also controls YAP/TAZ through regulatory elements, but decreases YAP transcriptional activity by recruiting co-repressor complexes (173). This shows that YAP/TAZ is capable of inducing gene expression, not only through direct interactions with transcription factors, but also through regulators which are in close proximity of nuclear YAP/TAZ. Which of these transcriptional regulators are activated in tumor ECs is currently still unclear.

### Canonical YAP/TAZ Effectors in Tumor Angiogenesis

Tumor ECs express a different transcriptional program compared to normal ECs (174). Many genes which are upregulated during physiological angiogenesis, are also upregulated in ECs in the TME (175, 176); for example increased VEGF-VEGFR2 signaling (177, 178). In addition, the expression of various genes involved in the interaction of ECs with immune cells are downregulated, suggesting that tumor-associated ECs play an important role in the immunotherapeutic resistance of the TME (179, 180). To understand how YAP/TAZ influences tumor angiogenesis, we need to consider the (potential) function of their downstream transcriptional targets.

Activation of YAP/TAZ induce the transcription of a canonical set of genes involved in proliferation, migration and cytoskeletal rearrangement and suppresses genes related to apoptosis in a variety of cell types (67, 94). In **Table 1** we summarize the currently known YAP/TAZ target genes and their possible involvement in (tumor) angiogenesis (**Table 1**). These genes were included as part of the endothelial YAP/TAZ target gene signature if detected in a minimum of three independent YAP/TAZ transcriptome studies, and have been confirmed at least once in an endothelial context (77, 79, 153, 158, 244, 245). For an overview of all YAP/TAZ target genes from these studies, see **Supplemental Table 1**.

**Table 1:** Selection of YAP/TAZ-regulated genes involved in (tumor) angiogenesis.

Gene	Function	Role in (tumor) angiogenesis	References
CYR61	Matricellular protein of the CCN family, regulates inflammation, wound healing, and fibrosis.	Promotes angiogenic processes through integrin αvβ3, which in turn activates VEGFR2 and downstream MAPK/PI3K signaling pathways.	(154, 181)
ANKRD1	Transcriptional effector, highly expressed in cardiac, and skeletal muscle and able to interact with transcription factors of different pathways.	ANKRD1-/- mouse have angiogenic impairments. ANKRD1 has been proposed to mediate angiogenesis through control of MMP-mediated ECM remodeling.	(182–184)
CTGF	Matricellular protein of the CCN family. A known mediator of fibrosis in multiple diseases. Cofactor required for TGFβ activity.	Activates VEGF signaling by aiding MMP-mediated cleavage of VEGF and prolongs VEGF signaling. Moreover, CTGF binding to integrin αvβ3 activates RhoA and promotes cell migration.	(185–187)
AMOTL2	Scaffold protein, acts as a link between VE-cadherin and contractile F-actin. AMOTL2 plays a regulatory role in the Hippo pathway.	AMOTL2 is a negative regulator of YAP/TAZ by preventing their nuclear translocation. The role of AMOTL2 in tumor angiogenesis is still debated, but it has been shown to promote proliferation and migration during angiogenesis and to promote tumorigenesis in specific tissues.	(57, 188, 189)
FGF2	Growth factor, interacts with FGF receptors and integrins. Plays an important role in the regulation of cell survival, division, differentiation, and migration.	Angiogenic growth factor implicated in tumor resistance to anti-VEGF tumor therapies. ECs are activated through MAPK and PI3K/Akt pathways, inducing MMP production, migration, and proliferation.	(190–192)
SLIT2	Secreted protein that acts as a guidance cue in cellular migration. Functions through the ROBO family as an inhibitor in processes neuronal migration and leukocyte chemotaxis.	SLIT2 has been implicated in tumor angiogenesis and shown to regulate endothelial migration. SLIT2 was found to suppress endothelial migration *in vitro*. Loss of SLIT2 resulted in decreased tumor vessel density in a tumor growth mouse model. Loss of endothelial SLIT2 in mouse models of breast and lung cancer suppressed tumor cell migration and metastatic events.	(193–196)
DLC1	Rho-GTPase-activating protein, controls RhoA inactivation, and regulates the actin cytoskeleton, cell shape, adhesion, migration, and proliferation.	DLC1 has a regulatory role in cell-contact inhibition of proliferation, EC migration and (tumor) angiogenesis. Interference of the DLC1-RhoA axis in knockout models disrupted cell migration and caused angiogenic defects.	(140, 197–199)
SERPINE1	Serine protease inhibitor regulating fibrinolysis through inhibition of tissue- and urokinase-type plasminogen activator. Major downstream effector of TGFβ signaling and upregulated in inflammatory, fibrotic, and thrombotic events.	SERPINE1 expression is positively correlated to tumorigenesis. Independent of its protease inhibitor activity, SERPINE1 regulates angiogenic related processes, such as matrix degradation, migration, proliferation, and cytoskeleton changes. Furthermore, inhibition of urokinase plasminogen activator was shown to inhibit angiogenesis.	(200–203)
CRIM1	(Putative) transmembrame protein containing IGF-binding domain. Plays a role in development and in different tissues by binding secreted growth factors.	CRIM1 plays a role in vascular development, capillary formation and angiogenesis by augmenting VEGF-A signaling through VEFGR2. In cancer, CRIM1 was shown to regulate cell adhesion and migration.	(204–207)
AXL	AXL is a receptor tyrosine kinase (RTK), that upon binding its ligand (growth factor GAS6) activates the PI3K/Akt pathway.	AXL plays a role in neovascularization by regulating EC proliferation, survival and migration. *In vivo* models demonstrated its importance in angiogenesis and tumor formation. AXL regulates angiogenesis by modulating Ang2 and DKK3 levels.	(208–212)
BIRC5	Mitotic spindle checkpoint gene, component of the mitotic apparatus, involved in chromosome alignment and segregation during mitosis and cytokinesis. Cell cycle dependent expression, promoting cell proliferation and inhibition of apoptosis.	Several studies found a correlation between tumor angiogenesis and BIRC5 expression. BIRC5 promotes endothelial proliferation and migration and inhibits apoptosis. BIRC5-dependent VEGF and FGF expression and modulation of the PI3K/Akt pathway are the proposed mechanisms for the angiogenic effect of BIRC5.	(213–217)
SHCBP1	Adaptor protein associated with cell surface receptors, involved in various signaling pathways, such as FGF, NF-κB, TGFβ-1/Smad, and β-catenin signaling.	SHCBP1 is upregulated in several cancers and promotes proliferation and migration. SHCBP1 overexpression increased VEGF expression and promoted angiogenesis through TGFβ/SMAD signaling.	(218–221)
SGK1	Serine/threonine-protein kinase, downstream effector of PI3K/mTORC2 signaling. Anti-apoptotic gene regulating cell growth, proliferation, survival, and migration.	Role in angiogenesis through phosphorylation of SGK1 target NDRG1 (also a transcriptional target of YAP/TAZ), which modulates NF-κB signaling and expression of VEGF. Activated NDRG1 induces expression of angiogenic CXC cytokines, such as IL-8. High microvessel density in tumors correlates with NDRG1 nuclear activity. Knockdown of SGK1/NDRG1 reduced tumor angiogenesis.	(222–225)
DDAH1	Role in the regulation of nitric oxide generation. Inhibits degradation of nitric oxide synthase.	Upregulated in tumor tissues, increases VEGF expression as a result of elevated nitric oxide levels and correlates with tumor growth and angiogenesis *in vivo*. Targeting of DDAH1 with a therapeutic compound resulted in regression of tumor size and tumor vasculature density.	(226–232)
TK1	Thymidine kinase, involved in cell division. Activity of the cytosolic enzyme is highest during the S-phase. Marker for proliferating cells.	TK1 is upregulated in tumor-associated ECs. Exact role of TK1 in tumor angiogenesis is still unknown, but it was found to act as both an angiogenic and angiostatic factor.	(233–235)
TGFB-2	Ligand activating the TGFβ receptor/SMAD pathway; activation of this pathway results in an increase in the deposition of ECM, angiogenesis, immunosuppression and alterations in cell adhesion.	Multifunctional protein, involved in regulation of angiogenesis. Implicated in tumor angiogenesis through activation of YAP/TAZ and upregulation of angiogenic factors. Also able to indirectly promote angiogenesis by reorganizing the ECM and promoting the immune system.	(164, 236)
ECT2	Rho GEF activating Rho-GTPases, like RhoA, RhoC, Rac1, and CDC42, plays a role in cell division.	During angiogenesis, ECT2 controls VEGF-induced activation of Rho-GTPases. Knockdown of ECT2 inhibits sprouting angiogenesis. During tumorigenesis, deregulated ECT2 drives tumor cell proliferation.	(237–240)
COL4A3	Subunit of type IV collagen, structural component of the basement membrane.	Expressed in angiogenic endothelial cultures and considered as an angiogenic factor. Upregulated in tumor tissue and COL4A3 expression levels are correlated to tumor progression. In contrast, Tumstatin (MMP product of COL4A3) suppresses angiogenesis.	(241–243)

**Table 1** highlights the most common YAP/TAZ target genes and provides an overview of the different mechanisms through which they regulate (tumor) angiogenesis. These genes were considered as common YAP/TAZ targets when found in a minimum of three independent YAP/TAZ transcriptome studies, and have been confirmed at least once in an endothelial cell context (77, 79, 153, 158, 244, 245). The full list of genes can be found in **Supplemental data file** 1. The variety of effectors through which YAP/TAZ affect tumor angiogenesis currently makes it difficult to discern the significance of each protein to the process. **Table 1** demonstrates the diversity of ways through which YAP/TAZ transcriptional targets are able to regulate tumor angiogenesis, e.g., as an extracellular matrix (ECM) component, a secreted factor, a modulator of a signaling pathway, a transcription factor in the nucleus or as a combination of such mechanisms. Furthermore, these YAP/TAZ targets are also expressed by other cells in the tumor microenvironment (TME), such as tumor cells and fibroblasts, and often work through a common mode of action to promote tumor angiogenesis.

The well-established YAP/TAZ transcriptional targets connective tissue growth factor (CTGF), Cysteine-rich angiogenic inducer 61 (CYR61) and Ankyrin Repeat Domain 1 (ANKRD1) were upregulated in all of the above-mentioned transcriptome studies. CTGF and CYR61 are part of the CCN protein family (246). CTGF is a known mediator of fibrosis in a wide range of diseases (247). During tumor progression, CTGF plays a role in ECM deposition and promotes proliferation and epithelial to mesenchymal transition (EMT) (159, 248). Stromal expression of CTGF increases micro-vessel density in prostate cancer xenografts (249). CTGF was found to promote angiogenesis by inducing VEGF-A secretion of TGFβ-stimulated fibroblasts (250). CTGF also promotes tumor angiogenesis through regulation of Ang2 (251).

The YAP/TAZ target CYR61 was demonstrated to promote angiogenesis and improve tissue perfusion in ischemic models (252). CYR61 null mice are embryonically lethal caused by vascular defects (253). CYR61 is expressed in angiogenic ECs at sites of neovascularization (254). Increased expression of CYR61 is found in many forms of cancer and is linked to an increase of size and vascularization of tumors (255–257). CYR61 promotes tumor angiogenesis through its interaction with integrin αvβ3, which in turn regulates endothelial adhesions, migration, proliferation, and activates VEGFR2 (154).

ANKRD1 is a transcriptional effector of YAP/TAZ. ANKRD1 has been shown to have an anti-inflammatory role through inhibition of NF-κB (182). ANKRD1 may act as a co-activator of the tumor suppressor p53, and the presence of p53 maintains the expression levels of ANKRD1 through a positive feedback loop (183, 258). In cancer ANKRD1 can be epigenetically inactivated (258), which may explain how such a well-established YAP/TAZ target does not attenuate YAP/TAZ-mediated tumorigenesis. ANKRD1 has been proposed to mediate angiogenesis through MMP-mediated ECM remodeling (184). ANKRD1 promotes angiogenesis after acute wounding of mouse skin (259, 260), but currently little is known about the potential role of ANKRD1 in tumor angiogenesis.

### Endothelial-Specific YAP/TAZ Angiogenic Effectors

YAP/TAZ also induce the expression of endothelial-specific genes to modulate the tumor vasculature. While not always being referred to as YAP/TAZ effectors in the literature, these pro-angiogenic proteins are readily investigated for their role in tumor angiogenesis and their potential as therapeutic targets.

#### Ephrin-Eph System

The Ephrin receptor genes EphA2-4 and EphB4 and Ephrin genes EFNB2-3 were found to be downstream targets of endothelial YAP/TAZ in developmental angiogenesis (79). Ephrins and Eph receptors are upregulated in almost all tumors and are considered as promising targets for cancer therapy (261). Interestingly, in YAP/TAZ transcriptome studies focusing on expression in cancer cell lines, the Ephrin family does not seem to be a prominent transcriptional target of YAP/TAZ (158, 244, 245), which indicates that Ephrins may be specifically derived from YAP/TAZ activation in the tumor endothelium.

The Eph receptors and Ephrin ligands are crucial for vascular specification during development *via* their signaling toward Rho-GTPases, cytoskeletal remodeling, and cell migration (262). Expression of the EphA2 receptor has been reported to promote tumor size and vascular density (263, 264). Genetic silencing or blocking activation of the EphA receptors attenuated tumor angiogenesis and decreased tumor vessel density (265, 266). Signaling through EphrinB2 and EphB4 has been directly associated with tumor angiogenesis and with tumor resistance to anti-angiogenic therapy (267, 268). Inhibition of EphB4 signaling through the use of soluble ligands, reduced the growth and vascularization of tumors (269). Another study showed that EphB4 suppresses sprouting angiogenesis and induces circumferential growth of blood vessels in tumor xenografts (270). Furthermore, the Ephrins and their receptors differ in expression levels between tumor types. For instance, EphA2 and EphB2 both promote tumor angiogenesis, but EphA2 is upregulated in prostate cancer, while EphB2 is downregulated (261). Before considering Ephrin signaling as therapeutic target to normalize the tumor vasculature, more understanding is needed of the downstream mechanisms of Eph receptors, as they induce vascularization in some cancers, but restrict tumor growth in others.

#### Angiopoietin-Tie System

Ang2 is a downstream transcriptional target of YAP/TAZ in ECs (75, 79). The ligands Angiopoietin-1 (Ang1) and Ang2, bind to Tie receptors and control the angiogenic activation of ECs to finely tune vascular development and homeostasis (271, 272). The Ang2 antagonist Ang1 is an important regulator of vessel maturation and Ang1-Tie2 signaling induces endothelial quiescence (272). Conversely, interaction of Ang2 with Tie2, results in disruption of EC monolayer integrity, making ECs more responsive to inflammatory and pro-angiogenic cytokines (273–275). Ang2 is produced by ECs and signals in an autocrine manner (274). Ang2 expression is induced in response to inflammatory cytokines, hypoxia, and haemodynamic forces (275, 276). Ang2 is highly expressed in ECs of remodeling vessels, indicating an important role for Ang2 in angiogenesis (275, 277). Interestingly, Ang2 may exert both pro- and anti-angiogenic functions. In the presence of VEGF, Ang2 has a pro-angiogenic effect on ECs, while in the absence of VEGF, apoptosis, and vessel regression is induced by Ang2 (278–281). Ang1 binding to Tie2 induces Tie2 autophosphorylation and downstream PI3K/Akt and ERK signaling pathways and inhibits NF-κB activation (271, 273). Moreover, Ang1-Tie2 signaling inhibits Ang2 expression, maintaining endothelial quiescence (273). Upregulation of Ang2 competes with Ang1-Tie2 signaling, resulting in destabilization of the vascular endothelium (273).

Ang2 is described to be a mediator of YAP-induced angiogenesis in mouse retinal vasculature (75, 84). Ang2 levels correlate with YAP activation in sprouting vessels in the retina (75). Supplementation of Ang2 rescues angiogenic defects in the retinal vasculature of YAP/TAZ knockdown mouse and marks Ang2 as a prominent downstream effector of YAP/TAZ-regulated angiogenesis (75). Blocking Ang2 was able to inhibit endothelial YAP-induced angiogenic sprouting (84). Ang2 is upregulated in several types of cancer and is a mediator of tumor angiogenesis (282). Interestingly, YAP and Ang2 association is also observed in the tumor vessels of melanoma (75). In astrocytomas, Ang2 upregulation was correlated with increased vascular growth and an abnormal tumor vasculature (283). Furthermore, angiogenic tumor vessels of human squamous cell carcinoma and skin carcinogenesis xenografts showed an upregulation of Ang2 (284). Interestingly, Ang1 overexpression inhibits tumor growth in these cancer models. The amount of vascularization was unchanged, however more pericyte coverage of the tumor vessels was observed, pointing toward vessel maturation (284, 285). Alternatively, in tumor ECs, Ang2 has been described to induce pro-angiogenic effects by triggering integrin adhesion signaling (286). Ang2 inhibition and Tie2 activation in experimental glioma models resulted in normalization of the tumor vessels, reduced hypoxia and acidosis in the TME, and reduced tumor growth (287). In experimental glioblastoma models, a combination of VEGF- and Ang2-inhibition, was also found to induce tumor vessel normalization (288). Overall, these findings indicate that the YAP/TAZ effector Ang2 might be a promising therapeutic target in cancer.

#### Fibroblast Growth Factor 2

Fibroblast growth factor 2 (FGF2) is a well-defined YAP/TAZ target expressed in both tumor cells and ECs (77, 79, 158, 244, 245). FGF2 signals through the FGF receptor (FGFR) tyrosine kinase family and induces a broad range of cellular functions, including proliferation, migration, and angiogenesis. Tumor-secreted FGF2, in conjunction with VEGF, promotes tumor angiogenesis (190, 191). The FGF2 pathway is being considered as an important angiogenic pathway involved in bypassing tumor resistance to anti-angiogenic therapies that target VEGF signaling (289, 290). Inhibiting FGF2-FGFR signaling in mice indeed improved tumor sensitivity to anti-VEGF therapy, suggesting that therapeutic strategies that target both growth factors could form a stronger anti-angiogenic intervention in cancer (192).

#### Deleted-in-Liver-Cancer 1

We recently discovered that Deleted-in-liver-cancer-1 (DLC1), a Rho GAP protein, functions as a direct target of YAP/TAZ in ECs (140). DLC1 is a potential tumor suppressor in various cancer types (291, 292). DLC1 is recruited to integrin-based adhesions through binding to the FA proteins talin, tensin and/or FAK (293, 294). DLC1 knock out mice are embryonically lethal, and the depletion of DLC1 leads to vascular defects (197, 295). DLC1 expression is upregulated by ECM stiffening and angiogenic VEGF signaling (140, 296), and it functions as a prominent target of YAP/TAZ by driving endothelial FA turnover, collective cell migration and sprouting angiogenesis (140). Perturbation of YAP/TAZ signaling and DLC1 levels affect endothelial contact inhibition and promote the development of angiosarcoma (198, 297). Intriguingly, depletion of DLC1 from normal epithelial cells, resulted in increased production of VEGF and upregulation of active HIF1α, suggesting that the absence of DLC1 in oncogenic cells can drive angiogenesis in a paracrine fashion (298). Whether DLC1 is an important target of YAP/TAZ in the tumor endothelium remains to be addressed.

#### CXCL Chemokines

Various chemokine CXC family members (e.g., CXCL1, CXCL6, CXCL12) are regulated by YAP/TAZ in the endothelium (79, 153). CXCL1, CXCL2, CXCL3, and CXCL8 are part of an angiogenic subset of the CXC family that regulate chemotaxis and angiogenesis through the GPCR CXCR2 (299). In physiological context, CXCL1 primarily targets ECs and neutrophils (299). However, CXCL1 and CXCR2 are also upregulated in different tumor tissues and induce tumor angiogenesis (300–303). It is thought that the inflammatory cytokines contribute to the TME by recruiting inflammatory cells and inducing stromal cell senescence (304–307).

### Are There Tumor Angiogenesis-Specific YAP/TAZ Effectors?

The YAP/TAZ-regulated transcriptome is tissue specific. In the endothelium, YAP/TAZ modulate angiogenesis by regulating angiogenic genes. Interestingly, Wang et al. compared RNA-sequencing data from endothelial-specific YAP/TAZ KO mice with that from VEGF-treated HUVECs. Intriguingly, the VEGF-regulated genes were enriched in the gene set that was downregulated upon YAP/TAZ depletion (79). This indicates that YAP/TAZ activation and VEGF signaling synergize to promote angiogenesis.

The transcriptome of tumor-associated ECs in human lung tumors were recently investigated at single-cell resolution (179, 180, 308). Tumor ECs upregulate genes involved in transcription, oxidative phosphorylation and glycolysis, whereas they suppress inflammatory genes. Interestingly, TEAD1 was found as one of the two transcription factors responsible for the tumor-associated endothelial phenotype (179). A large number of the upregulated genes in tumor-associated ECs as reported by Lambrechts et al. are also controlled by YAP/TAZ (**Supplemental table 1**). Furthermore, tumor-associated ECs strongly activate VEGF and Notch signaling (180), which is likely mediated by YAP/TAZ through crosstalk between these pathways. Treatment with anti-VEGF therapies converts the transcriptomic profile of tumor-associated ECs into a quiescent EC type (308), emphasizing that the activity of tumor ECs is sensitive to therapeutic interventions.

To address if tumor angiogenesis is promoted through specific genes, other than those employed during physiological angiogenesis, the gene expression profiles of ECs in resting liver, regenerating liver, and tumor-bearing liver were compared (233). This study confirmed the upregulation of established angiogenic genes involved in proliferation, such as Top2a, TK1, and Ki67. Incidentally, these genes have also been reported as downstream targets of YAP/TAZ (77, 79, 158, 244, 245). The study further identified two genes that were markedly upregulated during tumor angiogenesis and have been reported as YAP/TAZ targets, namely, SH2D5 (158) and Apelin (79).

SH2 domain containing protein 5, or SH2D5, is a transcriptional target of YAP/TAZ (158) and promotes tumor growth through interaction with transketolase, a regulator of the STAT3 signaling pathway (309). The STAT3 signaling pathway is essential during physiological and tumor angiogenesis (310). In tumors, sustained STAT3 signaling promotes VEGF expression and angiogenesis (311). Furthermore, STAT3 interacts with YAP to promote angiogenesis in a synergistic manner (109). By contrast, a recent paper described YAP to downregulate STAT3 activity and inhibit VEGF expression (312). By regulating STAT3 and VEGF signaling, SH2D5 might be involved in regulating YAP/TAZ activity during tumor angiogenesis.

Apelin is a secreted protein and its expression is regulated by endothelial YAP/TAZ (79). Apelin is required during vascular development (313) and controls initiation of angiogenesis (314). In tumors, increased levels of Apelin promote tumor angiogenesis (314–317). In addition, Apelin drives angiogenesis of lymphatic vessels (318). Interestingly, the GPCR of Apelin, is upregulated in ECs taking part in pathological angiogenesis (308). Because of the anti-angiogenic and anti-lymphangiogenic abilities of Apelin, Apelin has been proposed as a potential therapeutic target for tumor therapies (319).

## YAP/TAZ Sustain Tumor Angiogenesis Through Feedback Mechanisms

Physiological angiogenesis and tumor angiogenesis are largely driven by common mechanisms (174, 233). The tumor vasculature is however very different in its organization and function compared to healthy vasculature (10). YAP/TAZ promote the formation of a disorganized and dense tumor vasculature network and simultaneously prevent vessel maturation and specification by sustaining angiogenic signaling (40). In this section we provide an overview of the potential mechanisms through which endothelial YAP/TAZ sustain angiogenic signaling in the TME.

### Feedback Signals that Fine Tune YAP/TAZ

YAP/TAZ are regulated by genes that feedback on the upstream elements of the signaling pathway. For instance, the YAP/TAZ target NUAK2 sustains YAP/TAZ activity in breast cancer cells through inhibition of LATS1/2 (320). Pharmacological inhibition of NUAK2 reduces tumor growth in mice, indicating its activity is important to enforce tumorigenic YAP/TAZ signaling (320). By contrast, in collectively migrating ECs, NUAK2 provides negative feedback to YAP/TAZ by reducing actomyosin contractility (80), indicating that this YAP/TAZ target’s feedback function is tissue-dependent. Members of the AMOT family are known regulators of YAP/TAZ (321), and likely also play a role in regulating endothelial YAP/TAZ activity through feedback; since AMOT and AMOTL2 were identified as YAP/TAZ transcriptional targets in endothelial RNA-sequencing studies (77, 79). The p130 isoform of AMOT increases YAP transcriptional activity by binding to YAP and preventing LATS1-mediated YAP phosphorylation (322). In contrast, AMOTL2 inhibits YAP/TAZ activity by binding directly to YAP or to LATS1/2 kinases (57, 188). In most cancers the AMOT family promotes tumorigenesis, while in others its effect is inhibitory (321), indicating AMOT function to be tissue-specific. Altogether the involvement of the AMOT family in the regulation of YAP/TAZ activity and tumor angiogenesis is controversial and should be studied more closely in endothelial context. Interestingly, LATS1/2 kinases were found to phosphorylate AMOT and to inhibit angiogenesis in ECs, independent of YAP/TAZ transcriptional activity (165). These debated findings highlight the currently limited understanding behind the regulatory switches in the YAP/TAZ pathway.

YAP/TAZ activity is further defined by other transcriptional regulators. β-catenin binds to YAP/TAZ in the nucleus and the complex drives tumorigenesis in β-catenin-driven tumors (323). BIRC5 is one of the transcriptional targets of the β-catenin-YAP/TAZ complex that promotes tumorigenesis (323) and is also a target of endothelial YAP/TAZ (**Supplemental table 1**) (77). BIRC5 was found to upregulate VEGF expression in esophageal cancer cells (213), and may therefore potentially sustain tumor angiogenesis through VEGF signaling. The interaction of YAP/TAZ with β-catenin is regulated by the Wnt pathway: in the absence of Wnt activity, the APC/axin destruction complex degrades the β-catenin-YAP/TAZ complex (324, 325). Moreover, Wnt5a/b and Wnt3a promote YAP/TAZ activity through Rho-GTPase-mediated inactivation of LATS (326). YAP/TAZ, in turn, promote expression of DKK1, BMP4, and IGFBP4, inhibitors of the canonical Wnt/β-catenin signaling (326). Thus, the crosstalk events that take place between YAP/TAZ and Wnt signaling could be an interesting element in the sustained YAP/TAZ signaling in tumor growth and angiogenesis.

### YAP/TAZ-Triggered Positive Feedback Loops

#### Angiogenic Growth Factor Signaling

In angiogenic tissue, YAP/TAZ generate positive feedback by driving a transcriptional response that sustains VEGF-VEGFR2 signaling (79). VEGF signaling itself activates endothelial YAP/TAZ and promotes YAP-dependent STAT3 activation (79, 84, 109). In turn, activated STAT3 elevates VEGF expression (109, 311). YAP/TAZ further enhance VEGF signaling through the YAP/TAZ target CRIM1, a transmembrane receptor that is highly expressed in angiogenic ECs (204). CRIM1 interacts with VEGF, and promotes VEGFR2 phosphorylation (204, 205).

Several YAP/TAZ effectors amplify VEGF signaling downstream of the VEGFR. For instance, the YAP/TAZ effector Rho GEF ECT2 is essential for VEGF-mediated activation of RhoA and endothelial migration (237, 327). In addition, increased VEGF signaling induces MMP expression, which is important for matrix degradation and basement membrane remodeling during angiogenesis (328). MMPs are also upregulated by the YAP/TAZ transcriptional program (329, 330), and may in turn modulate VEGF signaling by controlling VEGFR2 expression (331, 332).

Active endothelial YAP/TAZ drive the expression of other angiogenic cytokines as well, such as FGF2, CXCL1 and TGFβ-2 (see **Supplemental table 1**). TGFβ-2 activates the TGFβ receptor/SMAD signaling axis to promote angiogenesis, whereas TGFβ-1 promotes angiogenesis through VEGF expression (164). Thus, endothelial YAP/TAZ activation sustains tumor angiogenesis by enhancing VEGF signaling and related angiogenic factors.

#### TME Stiffening and Cellular Contractility

YAP/TAZ promote a transcriptional program that increases TME stiffening and intracellular contractility, which in turn reinforces YAP/TAZ signaling (67, 97). Activation of YAP/TAZ in fibroblasts is well known to promote deposition of ECM proteins, secretion of MMPs and cross-linking enzymes (78, 97). Within tumors, the CAFs synthesize fibronectin and collagens, which are key constituents of the stiff tumor tissue and modulate tumor angiogenesis (78, 97). ECM stiffening induces endothelial YAP/TAZ signaling to promote angiogenic sprouting (131, 140). In addition, ECM stiffening and YAP/TAZ signaling jointly induce EndoMT, during which ECs undergo mesenchymal transformation and start to actively participate in TME stiffening and vascular remodeling (333). Along that line, YAP/TAZ signaling in cholangiocarcinoma cells promotes expression and deposition of MFAP5, which is an component of the elastin fibrils in the ECM and promotes tumor vasculature formation (102). The YAP/TAZ target SERPINE1 is another secreted factor that correlates with tumor progression and modulates angiogenesis by competing with ECM proteins for binding to integrins (200, 201).

Finally, YAP/TAZ also regulate expression of genes that increase cytoskeletal contractility to mechanoactivate and further enforce their signaling. Endothelial YAP/TAZ induce expression of well-known modulators of intracellular tension, including the Ephrin-Eph system and the Rho-GTPase family and their regulators, such as the Rho GAP DLC1 and Rho GEF ECT2 (77, 79, 140).

#### Inflammatory Factors

Inflammatory cytokines in the TME activate YAP/TAZ and sustain angiogenesis (334–338). Inflammatory cytokines activate AP-1 (339), which promotes oncogenic growth in conjunction with YAP/TAZ (171). Expression of the YAP/TAZ effector CYR61 is upregulated by inflammatory cytokines such as IL-1 and TNF-α (340). CYR61 enhances VEGFR2 activity and consequently endothelial YAP/TAZ activity through an integrin αvβ3-VEGFR2-MAPK/PI3K-YAP/TAZ axis and enhanced STAT3 activation (154). However, the details of the reciprocal regulatory mechanism between CYR61 and YAP/TAZ need further investigations, as another study described CYR61 to negatively regulate YAP/TAZ activity (341).

Inflammatory stimuli also trigger the release of the YAP/TAZ target Ang2. Interestingly, blocking the function of Ang2, impaired the interaction between ECs and immune cells and reduced tumor neovascularization (285), suggesting that Ang2 is one of the targets of the YAP/TAZ pathway that might be amenable to normalize the tumor vasculature. Ang2-Tie2 signaling weakens the junctional integrity between ECs (273, 274). The destabilization of cell-cell contacts is known to activate YAP/TAZ *via* the inhibition of LATS1/2 (62, 63). Moreover, the destabilization of VE-cadherin-based cell-cell junctions activates YAP and induces Ang2 expression (342), pointing toward a positive feedback loop between endothelial YAP and Ang2 signaling.

In conclusion, various factors of the TME activate YAP/TAZ in the endothelium. Activated YAP/TAZ promote angiogenesis through a subset of downstream effectors, while other targets further aggravate the TME conditions. **Figure 2** gives an overview of the positive feedback loops that likely sustain YAP/TAZ activation in the TME.

## Outlook

The oncogenes YAP/TAZ are interesting targets for cancer therapy as they play an essential role during tumor vascularization. YAP/TAZ have readily been investigated as therapeutic targets for the tumor stroma. Therapeutic interventions have focused on inhibiting YAP/TAZ by targeting upstream Hippo effectors or the YAP/TAZ-TEAD interaction; such strategies have been reviewed in great detail in recent years (343–345). One of the major challenges of targeting strategies is that YAP/TAZ modulate multiple signaling pathways and that completely blocking YAP/TAZ signaling likely has large side effects on tissue homeostasis (95). In this review, we highlight how YAP/TAZ is able to maintain a hyperactive endothelial state within the TME and how this leads to aberrant tumor angiogenesis. Targeting the transcriptional downstream targets that reinforce YAP/TAZ activity in the endothelium may provide an interesting approach to normalize tumor vasculature and improve the efficacy of cancer therapies.

When determining the role of transcriptional targets downstream of YAP/TAZ in a biological process, such as tumor angiogenesis, there are a few things to consider. First, besides promoting gene expression, YAP/TAZ are capable of silencing genes through recruitment of inhibitory co-factors. Therefore YAP/TAZ effectively downregulate a significant number of target genes (173, 346). Secondly, YAP and TAZ each control unique (as well as overlapping) transcriptomes (158) and could therefore affect tumor angiogenesis differently. While YAP and TAZ are considered closely related paralogs, structural differences between YAP and TAZ proteins likely affect their specific transcriptional activities, which may be relevant for cancer subtypes. Finally, endothelial YAP was shown to have a cytoplasmic function, from where it regulates EC migration through interaction with CDC42 in the mouse retinal neovasculature (83). Hence, future research aimed to understand the cytoplasmic role of YAP/TAZ during tumor angiogenesis could provide important new insights.

Finally, different microRNAs (miRNAs) and long noncoding RNAs (lncRNAs) have been reported to regulate YAP/TAZ activity (347–350). Surprisingly, little is known regarding noncoding RNAs downstream of YAP/TAZ. In renal cell carcinoma, expression of lncRNA lncARSR is increased in a YAP/TEAD-dependent manner. In turn, the binding of lncARSR to YAP prevented LATS-mediated phosphorylation and activated YAP (351). TAZ upregulates the miRNAs miR-224 and miR-135, which promote tumorigenesis through inhibition of tumor suppressor SMAD4 (352) and suppression of LATS kinases (353), respectively. Furthermore, YAP was found to downregulate the lncRNA MT1DP, a tumor suppressor that inhibits YAP expression (354). A recent review of Tu et al. nicely summarizes the crosstalk between lncRNAs and YAP/TAZ during tumorigenesis (355). The role of noncoding RNAs as effectors of YAP/TAZ in tumor angiogenesis and their promise as therapeutic application remains a topic to address in the nearby future.

Here, we have described upstream mechanisms of YAP/TAZ activation in (tumor) ECs and provided an overview of the downstream transcriptional effectors of YAP/TAZ that participate in the development of tumor vasculature. Many YAP/TAZ downstream targets drive a stiff, pro-inflammatory, hypoxic TME, creating a self-sustained positive loop of YAP/TAZ activity and tumor angiogenesis. Interfering with the crucial events in such YAP/TAZ signal amplification steps are expected to put a brake on pathological angiogenesis in tumors and help to inhibit tumor growth and progression.

## Author Contributions

All authors contributed to the article and approved the submitted version.

## Conflict of Interest

The authors declare that the research was conducted in the absence of any commercial or financial relationships that could be construed as a potential conflict of interest.

## Abbreviations

AMOT, Angiomotin; Ang1, Angiopoietin-1; Ang2, Angiopoietin-2; ANKRD1, Ankyrin repeat domain 1; CAFs, Cancer-associated fibroblast; CTGF, Connective tissue growth factor; CYR61, Cysteine-rich angiogenic inducer 61; ECM, Extracellular matrix; EndoMT, Endothelial to mesenchymal transition; ECs, Endothelial cells; FA, Focal adhesion; FAK, Focal Adhesion Kinase; FGFR, FGF receptor; FGF2, Fibroblast growth factor 2; GPCR, G-protein-coupled receptor; HIF1α, Hypoxia inducible transcription factor 1α; IFP, Interstitial fluid pressure; LATS, Large tumor suppressor kinase; MMPs, Matrix metalloproteases; RTK, Receptor tyrosine kinase; SAV1, Salvador family WW-domain-containing-protein-1; STAT3, Signal Transducer and Activator of Transcription-3; STK, Serine/threonine kinases; TAZ, Transcriptional Co-Activator With PDZ-binding Motif; TEAD, TEA domain family member; TME, Tumor microenvironment; VEGF, Vascular endothelial growth factor; VEGFR2, VEGF receptor 2; YAP, Yes-associated protein.
